# Measles and Pertussis outbreaks in England and Wales: a time-series analysis

**DOI:** 10.3310/nihropenres.13607.1

**Published:** 2024-10-07

**Authors:** Thomas Shepherd, Christian Mallen

**Affiliations:** 1 School of Medicine, Keele University, Staffordshire, England, ST5 5BG, UK

**Keywords:** Measles, Pertussis, Whooping cough, epidemiology, health surveillance, vaccination

## Abstract

**Background:**

Vaccine coverage for common infectious diseases such as Measles and Pertussis (also known as whooping cough) have been declining in England and Wales since 2014. Consequently, significant increases in Measles and Pertussis cases are observed in the community.

**Aim:**

To explore whether Google Trends offers a predictive utility as a health surveillance tool for Meases and Pertussis in England and Wales.

**Design and Setting:**

Google search data related to Measles and Pertussis, including common associated symptoms, were downloaded for 52 weeks from 07/01/2023 – 07/01/2024. Measles and Pertussis case data were retrieved from the weekly Notification of Infectious Disease (NOID) reports.

**Methods:**

The associations between searching and case data were explored using a time-series analyses, including cross-correlations, Prais-Winsten regression and joinpoint analysis.

**Results:**

Significant cross-correlations were found for Measles cases and “measles” searching ( *r=*.41) at a lag of -1 week. For Pertussis cases, searching for “whooping cough” ( *r*=.31), “cough” ( *r*=.39), “100 day cough” ( *r*.41) and “vomiting” ( *r*=.42) were significantly correlated at a lag of -3 to -2 weeks. In multivariable regression, “measles” remained significantly associated with Measles cases (β=.24, SE=.33, *p*=.02) as did “whooping cough” (β=.71, SE=.27, *p*=.01) and “cough” (β=1.99, SE=.54, *p*=.001) for pertussis.

**Conclusion:**

Increases in Measles and Pertussis cases follow increases in online searches for both diseases and selected respective symptoms. Further work is required to explore how GT can be used in conjunction with other health surveillance systems to monitor or even predict disease outbreaks, to better target public health interventions.

## Plain Language Summary

In England and Wales, declining vaccine coverage for Measles and Pertussis since 2014 has led to a resurgence of these diseases. This study explores the potential of Google Trends (GT) as a predictive health surveillance tool for monitoring Measles and Pertussis (Whooping cough) outbreaks. Google search data related to these diseases and their associated symptoms were analysed with disease cases data. Significant correlations were found between online search trends and disease cases, with Measles cases correlating with searches for "measles" and Pertussis cases with terms like "whooping cough" and "cough." These correlations suggest that increases in online searches precede rises in numbers of new diagnoses of measles and Pertussis, indicating GT could be useful in predicting outbreaks. Integrating GT with traditional surveillance systems could enhance early detection and response to disease outbreaks, enabling more targeted public health interventions. Further research is needed to explore the full potential and limitations of GT in disease surveillance and prediction.

## Introduction

Vaccinations are one of the most effective public health interventions ^1^. The UK’s childhood vaccination programme is offered up to the age of 5, protecting against 13 infectious diseases ^2^. Since 2014, there has been a decreasing trend in vaccine uptake, which has been exacerbated by the COVID-19 pandemic ^3^. In addition to vaccine uptake, the timeliness of the administration is also critical to prevent outbreaks, particularly for Measles and Pertussis, where evidence suggest delays in administration of the first dose are the biggest predictors of non-completion of the vaccination schedules ^4^. The number of children at 24 months who have completed their first dose of the Measles Mumps and Rubella (MMR) vaccine has recently fallen to 89.2%, down from 90.3% the previous year (with coverage falling below 90% in 61 of the 149 Local Authorities) and those completing the second dose by age 5 down nearly 1% from the previous year ^5^. Between 2022–2023 the prenatal vaccination rate for Pertussis was 60.7%, representing a 4% drop from the year ending 2022 and 7.1% decrease from 2021 ^6^. Vaccine rates for Measles and Pertussis in the UK are below the 95% target set by the World Health Organisation ^7^.

As a consequence of decreasing vaccination rates, waning vaccine protection and the high transmissibility of both Measles and Pertussis, cases have been steadily rising in England and Wales ^8^. In the last 6 months of 2023 there were 1459 cases of Pertussis and 999 cases of Measles, compared to 428 and 658 in the first 6 months, respectively. These data are also substantially higher compared to the same period in 2022 (337 and 397) and in 2021 (296 and 249) ^9^.

Early identification of outbreaks is critical. Firstly, to implement targeted interventions, preventing further transmission, protecting vulnerable populations, and reducing mortality and morbidity (including reducing secondary infection risk) ^10^. Secondly, it provides a platform for communication and public awareness efforts. Rapid dissemination of information to healthcare providers, communities, and the public is critical for promoting preventative measures, encouraging vaccination, dispelling misinformation - contributing to the broader goal of achieving and maintaining population immunity ^11^. There is a critical need to explore additional mechanisms to traditional health surveillance techniques, particularly those operating in real time, to monitor cases or even predict outbreaks.

There is a growing body of research evidencing of the utility of online health information searching (OHIS) for health research across several domains, exploring; the impact of major disease awareness programmes ^12– 14^ the relationship between search data and disease burden measures ^15^ and how outbreak notifications impact OHIS ^16^. OHIS has also been used to predict and monitor disease outbreaks in combination with traditional health surveillance systems and has been shown to accurately predict cases of COVID-19 ^17^, HIV ^18^, Zika ^19^, self-harm ^20^ and influenza ^21^. However, OHIS has not been used to predict outbreaks of childhood infectious diseases, such as Measles and Pertussis in the UK.

In this exploratory time-series study, we explored relationships between OHIS for Measles and Pertussis and cases in the context of the current outbreaks in England and Wales, to investigate whether Google Trends (GT) offers a predictive utility as a health surveillance tool.

## Methods

### Patient and Public Involvement

No patients were involved in the study. Public members contributed to the conception of the study as part of wider Patient and Public Involvement group discussion around vaccine preventable, childhood infectious diseases. Members discussed the common use of internet searching for signs and symptoms in their children prior to seeking healthcare. Members further stressed the importance of this facility as a key trigger for subsequent healthcare seeking and wider recognition of childhood infectious diseases.

### Google Trends data

Anonymised search data can be retrieved using the GT platform which is free and publicly accessible. Data is provided as a Relative Search Volume (RSV), whereby searches are scaled between 0–100, where 100 represents its maximum popularity in a specified geography and time period ^22^. Duplicate searches, those including special characters or with very low search volumes are omitted. To ensure transparency and the reproducibility of GT-based research, reporting guidelines recommended by Nuti *et al*. were adhered to ^23^.

### Measles and Pertussis cases

In England and Wales, Measles and Pertussis are both notifiable diseases; clinical testing laboratories and medical practitioners are lawfully required to inform the UK Health Security Agency (UKHSA) of microbiologically or symptomatically suspected cases ^24^. Measles and Pertussis case data were retrieved from the weekly Notification of Infectious Disease (NOID) reports published on the government website on 22 January 2024 ^25^. Cases data were extracted from 01 January 2023 (week 1) to the week of 07 January 2024 (week 52).

### Search variables

Search terms were common synonyms and symptoms of Measle and Pertussis as detailed on the UK National Health Service (NHS) website ^26, 27^. Search terms for Measles were “measles”, “fever”, “runny nose”, “blocked nose”, “cough”, “sneezing”, “watery eyes”, sore eyes”, “rash” and “spots”. For Pertussis, search terms were “pertussis”, “whooping cough”, “cough”, “100 day cough”, “vomiting”. Search terms were entered as ‘topics’. GT describes a topic as ‘a group of terms that share the same concept in any language’. The topic feature encompasses searches for relevant subthemes. For instance, “Measles” includes Google Trends data for the search input “Measles symptoms” ^22^. Additionally, the topic feature encompasses linguistic variations, incorporating searches conducted by non-English speakers or in an alternative language. The category filter was set to ‘all’. Data were downloaded from GT 22 January 2024. No ethical approvals were required for this study as all data are publicly available and anonymised.

### Statistical analysis

Time-series cross-correlations were used to explore the relationship between search terms and case data over time. The advantage of using cross-correlations is that they account for the time dependence (lag) in the relationship between two time-series variables. In this case, significant cross-correlation at a lag (week) with a minus value, would suggest searching might predict case data in the subsequent weeks (determined by lag value). Conversely, positive correlations the positive lags will indicate that cases may also predict OHIS, that many weeks later. 

To further explore the association between searching and cases, a Prais-Winsten regression analysis was conducted due to the likely presence of serial correlation in the time series data. Variables were selected for the regression analysis if significantly correlated, as determined by Spearman’s Rank test. Univariable and multivariable models were fitted to better understand the predictive utility of respective search terms. Regression results are presented as coefficients and their standard error (SE). A *p* value of less than .05 was considered significant.

Temporal trends of cases and significantly associated search terms as determined by the Prais-Winsten regression were also explored using a Joinpoint regression analysis. A Weighted Bayesian Information Criterion (WBIC) was used to apply a single joinpoint to case and search term data to identify the week with the most significant trend change. The identification of a joinpoint in searching data in a week that precedes the joinpoint in case data would also be indicative of the predative utility. Analyses were conducted using Statistical Package for the Social Sciences (version 26.0) ^28^ and the Joinpoint Analysis Program (version 5.0.2) ^29^.

## Results

### Cases and Google Trends data

**Figure 1.**
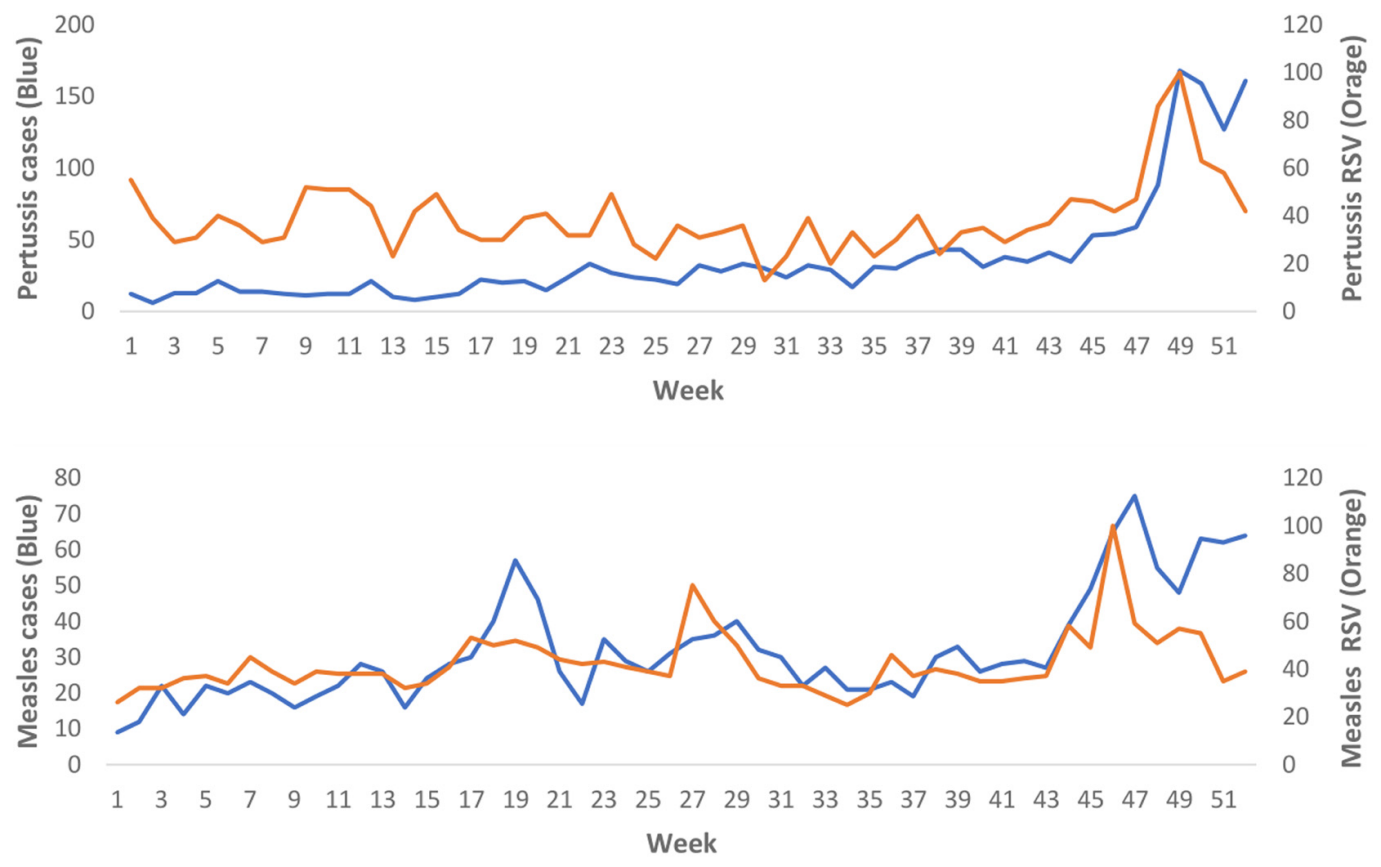
Measles and Pertussis cases with realtive search volumes for the study period.

**Figure 2.**
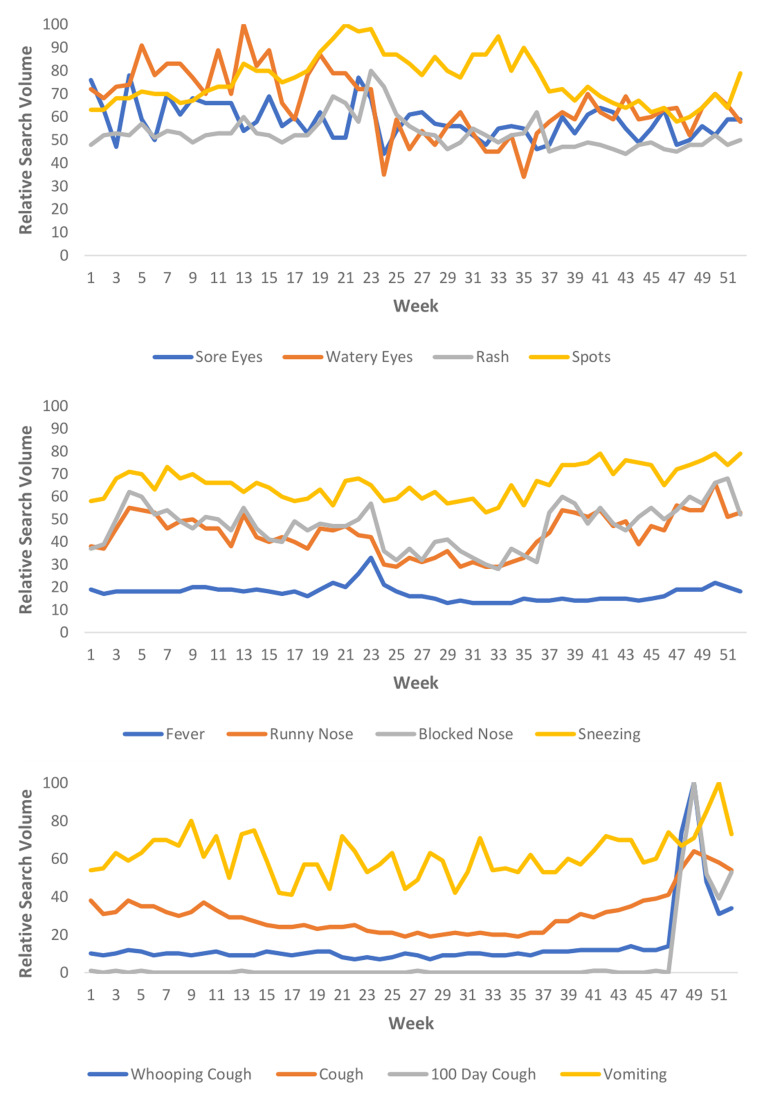
Relative Search Volume for all search terms across the study period.

The total number of cases for Measles and Pertussis over the 52-week study period (01/01/2023 – 07/01/2024) and the average number of cases per week are presented in Table 1. Measles and Pertussis cases with the RSV for “Measles” and “Pertussis” are illustrated in Figure 1. The RSV for other search terms across the study period are presented in Figure 2.

**Table 1.** Measles and Pertussis cases for the study period.

	Number of cases	Mean cases per week (SD)	Range (min-max)
Measles	1657	31.87 (15.14)	(9-75)
Pertussis	1887	36.29 (37.63)	(6-168)

SD, Standard Deviation’ Min, Minimum; Max, Maximum.

### Time series cross-correlation analysis

Linear association between case data and searching was explored using time-series cross correlations as shown in Table 2. For Measles cases, searching for “measles”, was significantly positively correlated from a lag of -1 weeks, through to +3 weeks. “Cough” searching was positively correlated from -4 weeks to cases. “Runny nose”, “blocked nose” and “sore eyes”, showed weak but positive correlations at lag 0. For Pertussis cases, “Pertussis” searching was significantly and positively correlated at -1 weeks onwards. Searching for “whopping cough”, “cough” and “100 day cough” were all significantly positively correlated with cases from -3 weeks to +3 weeks, and “vomiting” from -2 weeks to +2 weeks, respectively.

**Table 2.** Cross-correlation coefficients for search terms and cases of Measles and Pertussis.

Disease	Search Term	Lag										
		-5	-4	-3	-2	-1	0	+1	+2	+3	+4	+5
Measles												
	Measles	-.03	.07	.13	.27	**.41**	**.62**	**.67**	**.56**	**.41**	.30	.21
	Fever	.13	**.31**	.29	.15	.08	.10	.03	-.04	-.04	-.03	-.06
	Runny nose	-.04	.06	.17	.21	.27	**.33**	.23	.23	.18	.20	.21
	Blocked nose	.05	.15	.18	.18	.26	**.37**	**.36**	.27	.17	.13	.18
	Sneezing	.11	.15	.22	.26	.24	.32	.31	.36	.35	.36	.39
	Cough	.29	**.40**	**.45**	**.51**	**.52**	**.51**	**.49**	**.44**	**.32**	.21	.14
	Sore eyes	-.16	-.04	-.11	-.12	-.29	**-.30**	-.27	-.26	-.26	-.09	-.07
	Watery eyes	**-.33**	-.27	-.16	-.13	-.17	-.13	-.15	-.19	-.23	-.09	-.01
	Rash	.13	.10	.02	-.07	-.11	-.13	-.29	-.26	-.23	-.23	-.17
	Spots	-.22	-.30	**.32**	**.32**	.30	-.17	-.12	.02	.04	.07	.10
Pertussis												
	Pertussis	.11	.14	.18	.29	**.41**	**.59**	**.70**	**.58**	.50	.30	.10
	Whopping cough	.14	.20	**.31**	**.39**	**.53**	**.80**	**.83**	**.69**	**.57**	.28	.04
	Cough	.20	.25	**.39**	**.53**	**.66**	**.78**	**.73**	**.60**	**.47**	**.32**	.20
	100 day cough	.16	.24	**.41**	**.51**	**.65**	**.89**	**.80**	**.61**	**.47**	.18	-.02
	Vomiting	.11	.15	.25	**.42**	**.48**	**.47**	**.46**	**.28**	.17	.10	.12

Note. Coefficients in bold indicate statistical significance as determined by cross-correlation coefficients exceeding the upper confidence limit.

### Prais-Winsten regression

Spearman’s Rank correlations between cases and search terms are provided in Table 3 and Table 4. Search terms significantly correlated to respective cases were included in the regression analyses. Univariable and multivariable regression analyses (see Table 5) identified individual and combined effect of search terms on Measles and Pertussis case numbers. In the Univariable analysis the search term “Measles” was significantly positively associated with Measles cases (β =.22, SE=.07, *p*=.002). Search terms “Whooping cough” (β=.85, SE=.12, *p*<.001), “cough” (β=.62, SE=.21, *p*=.004) and “100 day cough” (β=1.05, SE=.11, *p*<.001) all showed significant positive associations with Pertussis cases. Due to a high degree of correlation between “whooping cough” and “100 day cough” ( *r*=.97), only “whooping cough” was included in the multivariable analysis on the basis of its greater average RSV across the study period (13 vs 2, respectively). Analysis revealed significant association between the search term “measles” and measles cases (β=.22, SE=.10, *p*=.03). Pertussis cases were significantly positively associated with “whooping cough (β=.89, SE=.21, *p*<.001) and “cough” (β=2.56, SE=.45, *p*<.001).

**Table 3.** Spearman’s Rank correlation coefficient matrix for Measles cases and all Measles-related search terms.

	Measles cases	Measles	Fever	Runny nose	Blocked nose	Sore eyes	Watery eyes	Rash	Spots	Sneezing
Measles cases		***r*=.62,** **p<.001**	*r*=.10, p=.49	***r*=.34,** **p=.02**	***r*=.37,** **p=.007**	***r*=-.30,** **p=.03**	*r*=-.13, p=.36	*r*=.13, p=.37	*r*=-.17, p=.23	***r=*.32,** ***p=*.02**
Measles	***r*=.62,** **p<.001**		*r*=.07, p=.67	*r*=.10, p=.46	*r*=.16, *p*=.27	*r*=-.09, *p*=.56	*r*=-.07, *p*=.65	*r*=-.05, *p*=.72	*r*=-.12, *p*=.39	r=.08, p=.57
Fever	*r*=.10, p=.49	*r*=.07, *p*=.67		*r*=.28, *p*=.05	***r=.*39**, ***p*=.004**	***r*=.34,** ***p*=.01**	*r* **=.39,** ***p*=.004**	***r*=.62,** ***p*<.001**	*r*=.27, *p*=.11	*r*=.10, *p*=.49
Runny nose	***r*=.34,** **p=.02**	*r*=.10, *p*=.46	*r*=.28, *p*=.05		***r*=.87** ***p*<.001**	*r*=.10, *p*=.50	***r=*.49**, ***p*<.001**	*r*=-.22, *p*=.12	***r*=-.51,** ***p*<.001**	***r*=.73,** ***p*<.001**
Blocked nose	***r*=.37,** **p=.007**	*r*=.16, *p*=.27	***r=.*39**, ***p*=.004**	***r*=.87** ***p*<.001**		*r*=.16, *p*=.27	***r*=.44,** ***p*=.001**	*r*=-.14, *p*=.33	***r*=-46,** ***p*=.001**	***r*=.73,** ***p*<.001**
Sore eyes	***r*=-.30,** **p=.03**	*r*=-.09, *p*=.56	***r*=.34,** ***p*=.01**	*r*=.10, *p*=.50	*r*=.16, *p*=.27		***r*=.37,** ***p*=.007**	*r*=-.05, *p*.72	*r*=-.07, *p*=.61	*r*=.10, *p*=.46
Watery eyes	*r*=-.13, p=.36	*r*=-.07, *p*=.65	***r*=.39,** ***p*=.004**	***r=*.49**, ***p*<.001**	***r*=.44,** ***p*=.001**	***r*=.37,** ***p*=.007**		*r*=.12, *p*=.41	*r*=-.12, *p*=.39	*r*=.20 *p*=.16
Rash	*r*=.13, p=.37	*r*=-.05, *p*=.72	*r* **=.62,** ***p*<.001**	*r*=-.22, *p*=.12	*r*=-.14, *p*=.33	*r*=-.05, *p=*.72	*r*=.12, *p*=.41		***r*=.69,** ***p*<.001**	***r*=-.31,** ***p*=.03**
Spots	*r*=-.17, p=.23	*r*=-.12, *p*=.39	*r*=.27, *p*=.11	***r*=-.51,** ***p*<.001**	***r*=-46,** ***p*=.001**	*r*=-.07, *p*=.61	*r*=-.12, *p*=.39	***r*=.69,** ***p*<.001**		***r*=-.54,** ***p*<.001**
Sneezing	***r=*.32,** ***p=*.02**	r=.08, p=.57	*r*=.10, *p*=.49	***r*=.78,** ***p*<.001**	***r*=.73,** ***p*<.001**	*r*=.10, *p*=.46	*r*=.20 *p*=.16	***r*=-.31,** ***p*=.03**	***r*=-.54,** *p* **<.001**	

Significant correlations are highlighted in bold.

**Table 4.** Spearman’s Rank correlation coefficient matrix for Pertussis cases and all Pertussis-related search terms.

	Pertussis cases	Pertussis	Whooping cough	100 day cough	Cough	Vomiting
Pertussis cases		***r*=.59,** ***p*<.001**	***r=*.80,** ***p*<.001**	***r=*.89,** ***p*<.001**	***r=*.78,** ***p*<.001**	***r*=.47,** ***p*<.001**
Pertussis	***r*=.59,** ***p*<.001**		***r*=.81,** ***p*<.001**	***r*=.75,** ***p*<.001**	***r=*.75,** ***p*<.001**	***r*=.40,** ***p*=.003**
Whooping cough	***r=*.80,** ***p*<.001**	***r*=.81,** ***p*<.001**		***r*=.96,** ***p*<.001**	***r*=.77,** ***p*<.001**	***r*=.97,** ***p*<.001**
100 day cough	***r=*.89,** ***p*<.001**	***r*=.75,** ***p*<.001**	***r*=.96,** ***p*<.001**		***r*=.80,** ***p*<.001**	***r=*.41,** ***p*=.003**
Cough	***r=*.78,** ***p*<.001**	***r=*.75,** ***p*<.001**	***r*=.77,** ***p*<.001**	***r*=.80,** ***p*<.001**		***r*=.61,** ***p*<.001**
Vomiting	***r*=.47,** ***p*<.001**	***r*=.40,** ***p*=.003**	***r*=.97,** ***p*<.001**	***r=*.41,** ***p*=.003**	***r*=.61,** ***p*<.001**	

Significant correlations are highlighted in bold.

**Table 5.** Prais-Winsten regression analysis of correlated search terms and case data.

Disease	Search term	Univariable regression			Multivariable regression		
		β	SE	*p-*value	β	SE	*p-*value
Measles							
	Measles	.22	.10	***p*=.03**	.24	.33	***p*=.02**
	Runny nose	.41	.20	*P*=.05	.42	.24	*p*=.09
	Blocked nose	.25	.17	*p*=.16	.15	.12	*p*=.46
	Sneezing	.11	.24	*P*=.64	-.01	.25	*p*=.27
	Cough	-.16	.30	*p*=.60	-.32	-.16	*P*=.27
Pertussis							
	Pertussis	.29	.21	*p*=.14	-.36	.22	*p*=.10
	Whooping cough	.89	.19	***p*<.001**	.71	.27	***p*=.01**
	Cough	2.56	.45	***p<*.001**	1.99	.54	***p*=.001**
	100 day cough	1.09	.15	***p*<.001**	-	-	-
	Vomiting	-.14	.21	*p*=.52	-.01	.20	*P*=.95

SE, Standard Error; Significant results are highlighted in bold.

### Joinpoint analysis

Joinpoint analysis (see Table 6) revealed the most significant increase in trends for Measles cases at week 42, however the significant joinpoint for “measles” searching was identified at week 39. For Pertussis, the joinpoint was found to be week 45, as was the joinpoint for “whooping cough”, but for “cough” the significant joinpoint was identified at week 35.

**Table 6.** Joinpoint regression analysis outcomes illustrating the weekly position of the most significant change in trend.

Joinpoint regression analysis					
Variable	WBIC	MSE	SSE	Week	95% confidence interval
Measles cases	1.11	1.36	56.46	42	39-44
“Measles”	1.17	2.56	61.78	39	29-49
Pertussis cases	1.02	2.10	50.78	45	43-47
“Whooping cough”	1.03	2.16	52.75	45	32-47
“Cough”	-0.90	0.30	7360	35	33-37

WBIC, Weighted Bayesian Information Criterion; MSE, Mean Squared Error; SSE, Sum of Squared Error.

## Discussion

We explored the relationship between OHIS and case data for Measles and Pertussis to assess whether the use of key search terms could support public health surveillance or even predict outbreaks of both diseases. Our analyses strongly suggests that GT provides a predictive utility for the increases in cases for Measles and Pertussis in advance of diagnosis based on disease and symptom searching. For Measles cases, the search term “measles” significantly predicted subsequent cases 1 week later, searching for “spots” offered week predictive utility for cases 3 weeks later. For Pertussis cases, the search term “Pertussis” offered a less robust association with Pertussis cases, however this is likely attributable to it being a less known term in the community. However, the more informal terms “whooping cough” and “100 day cough” showed a much more significant predictive relationship, but the strongest association was found for “cough”. Increased searching for cough was significantly predictive of Pertussis cases 3 weeks later and remained the most significant predictor of cases in multivariable analysis.

There are limitations that need to be considered. Firstly, other events, such as news reports may have impacted searching trends, particularly in the context of Measles and Pertussis outbreaks, however time series analysis in this study suggests that OHIS was occurring before the diagnostic and disease notification process. Secondly, this study only used Google searching, missing out on searching using other platforms, however Google remains the most frequently used search engine in the UK (93.69% of devices) ^30^. Thirdly, calculations applied to obtain RSVs are not publicly available and applied algorithms may obscure the observed trends, however, previous evidence has shown the data to accurately predict other infectious disease outbreaks ^18^ and be comparable to the United States Centre for Disease Control in predicting the timing of influenza outbreaks in the US ^21^. Currently, the provision of location specific searching provided by GT is inadequate for the UK, so data pertain to the national level only. Being able to divide search data geographically, perhaps by county or Integrated Care Board would enhance the predictive utility of GT and elucidate the locations of cluster outbreaks that require urgent response. This would allow for the inclusion of vernacular and terminology specific to different geographies. Finally, this study is retrospective, protocols using GT for health surveillance techniques and outbreak monitoring in real time need to be developed and tested. 

This is the first study to explore the predictive utility of GT for measles and Pertussis in England and Wales, however findings are consistent with previous evidence supporting the health surveillance utility of OHIS data. Samaras *et al.* employed a gamma distribution model showing how OHIS predicts outbreaks of Scarlet Fever 5 weeks before the peak using symptoms and scarlet fever associated search terms ^31^. Furthermore, GT data was utilised by Prasanth et al alongside modelling techniques to accurately forecast COVID-19 incidence, cumulative cases, and deaths at the national level during the pandemic ^32^. GT data has also been used to accurately predict Norovirus cases in the UK and more accurately than other real time data searching sources, including Wikipedia ^33^. Internationally, GT data has been used to predict infectious disease outbreaks at more local levels, forecasting malaria, dengue fever, chikungunya, and enteric fever outbreaks in two districts in India ^34^. In the US, GT has been shown to accurately predict HIV case numbers ^18^ and mpox outbreaks by state ^35^ as well as influenza cases and hospitalisations ^36^.

In addition to changes in trends for disease cases reflecting earlier changes observed in OHIS, the converse was also shown in our time series analysis, where OHIS for Measles and Pertussis were subsequently predicted by increases in cases. This is likely attributable firstly to increasing cases over time and secondly, as those receiving diagnoses begin to search for disease-related or symptom management information ^37^. This is an important finding illustrating the need for the provision of high-quality, evidence-based public health related information to support communities with transmission prevention techniques, healthcare seeking guidance and to prevent the spread and impact of misinformation ^16^. Additionally, it is expected that subsequent engagement with Measles and Pertussis topics take places via alternative digital platforms. Mahroum et al prosed a stimulus-awareness-activism framework, where surges in OHIS are followed by further online and offline discourse and has been evidenced by increased Twitter/X discussion on Chikengunya following outbreaks ^38^.

Not all symptom searching offered the same predictive utility for example “fever” searches offered little predictive utility for Measles cases. This is an important finding as this may reflect how different symptoms present at different times across the course of the disease and possibly how more troubling symptoms lead to higher levels of searching ^39^. Future work exploring GT as a health surveillance tool must identify the symptoms for which searching most accurately associates with case data.

Outside of increasing vaccine uptake in the community for Measles and Pertussis, additional real time surveillance tools to increase the likelihood of the early identification of case increases for Measles and Pertussis is of critical importance. This study suggests OHIS data, as provided by GT, can work as an alternative or adjunct to more traditional health surveillance systems (Royal College of General Practice Weekly Returns, NOIDs etc). Early detection allows for targeted interventions, preventing further transmission, protecting vulnerable populations in the community, and reducing mortality and morbidity ^10^.

## Conclusion

Our findings suggest that trends in online health information seeking for Measles and Pertussis precede trends in cases in England and Wales, indicating that GT may provide an additional, real time data source with predictive utility in detecting outbreaks of Measles and Pertussis. Further work is required to explore how GT data can be used in conjunction with other, traditional health surveillance systems to monitor or even predict disease outbreaks, to better target public health interventions.

## Ethics and consent statement

No Ethics and consent required for the performed study

## Data Availability

Figshare.com. Measles and Pertussis case and searching data. https://doi.org/10.6084/m9.figshare.27014038.v1 ^40^. The project contains the following underlying data: “MeaslesPertussisDataset”. (Measles and Pertussis case and Google Trends symptom search data). Data are available under the terms of the Creative Commons Zero "No rights reserved" data waiver (CC0 4.0 Public domain dedication).
