# Supplementary material for: Measles and Pertussis outbreaks in England and Wales: a time-series analysis
Source: NIHR Open Res. 2024 Oct 7;4:56. [Version 1] doi: 10.3310/nihropenres.13607.1 (PMC12521895; doi:10.3310/nihropenres.13607.1)
Supplement: Peer review history

# Reviewer report: Reviewer response for version 1

**DOI:** 10.3310/nihropenres.14772.r37264

**Published:** 2025-10-14

**Authors:** M Carolina Danovaro-Holliday

**Affiliations:** 1 World Health Organization, Geneva, Switzerland

The manuscript “Measles and Pertussis outbreaks in England and Wales: a time-series analysis” presents a simple and elegant time analysis of the relationship between the occurrence of measles and pertussis and Google searches of words related to those diseases.

 The analysis is sound, and the write-up is clear. My comments are mainly noting some typos and for clarity to non-UK audiences.

 Throughout: it is not necessary to capitalize disease names, so pertussis can be written with a small p.

 Comments by section

 Abstract

 Background – please clarify that increases in both measles and pertussis cases started to occur in late 2023 and continued through 2024

 Aim – typo in the word measles (it says “Meases”)

 Design and Setting – revise dates, a bit different from what is in the text. Also, please use month names, as date formats are not universal (is it January or July?)

 Conclusion – use of acronym GT - Spell-out or qualify when first using the term Google Trends

 Introduction - May want to mention schedule for Tdap and DTaP containing vaccines, plus for MMR in the UK. How many boosters, maternal vaccination?

 Methods - Patient and Public Involvement - Give more info on this "Patient and Public Involvement group", what is that?

 Google Trends data – what is the geographical scope? Is it only England and Wales? From the discussion, it seems that it may be for all of the UK.

 Measles and Pertussis cases – are the dates listed here and in the graphs, dates of reporting/notification or dates of onset? Please clarify.

 Discussion – please correct “United States Centre for Disease Control” to the correct name “Centers for Disease Control and Prevention”

 Discussion – typo in chikungunya (it says “Chikengunya” right before reference 38)

 References – check consistency, some have “Accessed on…” in parenthesis and while others don’t, some come after periods, and other after semicolons.

Is the work clearly and accurately presented and does it cite the current literature?

Yes

If applicable, is the statistical analysis and its interpretation appropriate?

Yes

Are all the source data underlying the results available to ensure full reproducibility?

Yes

Is the study design appropriate and is the work technically sound?

Yes

Are the conclusions drawn adequately supported by the results?

Yes

Are sufficient details of methods and analysis provided to allow replication by others?

Yes

Reviewer Expertise:

Immunization, disease surveillance, measles, vaccine-preventable diseases

I confirm that I have read this submission and believe that I have an appropriate level of expertise to confirm that it is of an acceptable scientific standard.

# Reviewer report: Reviewer response for version 1

**DOI:** 10.3310/nihropenres.14772.r33526

**Published:** 2024-11-22

**Authors:** Pedro Plans-Rubió

**Affiliations:** 1 College of Physicians of Barcelona, Barcelona, Spain

The study assessed the potential of Google Trends as a surveillance tool for detecting measles and pertussis (whooping cough) outbreaks. The study assessed the correlation between Google search data related to measles and pertussis and their weekly reported cases during the 07/01/2023 − 07/01/2024 period.

 Google search data related to Measles and Pertussis, including common associated symptoms, were downloaded. The associations between searching and case data were assessed using a time-series analyses, including cross-correlations, Prais-Winsten regression and join point analysis.

 The study found significant correlations between online Google search trends for measles and whooping cough with measles and pertussis cases, respectively. In multivariable regression analysis, Google search for “measles” was significantly associated with measles cases (β=.24, SE=.33, p=.02), and Google search for “whooping cough” (β=.71, SE=.27, p=.01) and “cough” (β=1.99, SE=.54, p=.001) were significantly associated with pertussis cases.

 The study added new data on the use of non-traditional measles and pertussis surveillance systems for the early detection and management of measles and pertussis outbreaks. Traditional measles and pertussis surveillance systems are based on collecting and assessing reported cases. However, they are challenged by the sudden appearance of measles and pertussis outbreaks.

 The worldwide spread of interned and smartphones has made it possible to collect and assess health-related information in real time. The correlations found in the study suggest that increases in online searches precede rises in numbers of new diagnoses of measles and pertussis. The results found in this study for measles and pertussis using Google queries  were consistent with the results found in prior studies using web queries for other infectious diseases, such as influenza, COVID-19 and scarlet fever. Integrating internet search, using Google and other internet platforms, with traditional surveillance systems could improve outbreak detection systems, making it possible to develop early targeted public health interventions to protect vulnerable populations and to reduce disease transmission.

 Further research is necessary to confirm the results obtained using the Google platform for longer study periods, other internet platforms and different disease/outbreak detection and reporting systems.

Is the work clearly and accurately presented and does it cite the current literature?

Yes

If applicable, is the statistical analysis and its interpretation appropriate?

Yes

Are all the source data underlying the results available to ensure full reproducibility?

Yes

Is the study design appropriate and is the work technically sound?

Yes

Are the conclusions drawn adequately supported by the results?

Yes

Are sufficient details of methods and analysis provided to allow replication by others?

Yes

Reviewer Expertise:

Evaluative studies, Vaccines, Vaccination strategies, Health Economics, Preventive Medicine, Public Health, Epidemiology, Influenza Surveillance, Syndromic Surveillance methods

I confirm that I have read this submission and believe that I have an appropriate level of expertise to confirm that it is of an acceptable scientific standard.
